# Assessment of Inter-Laboratory Variation in the Characterization and Analysis of the Mucosal Microbiota in Crohn’s Disease and Ulcerative Colitis

**DOI:** 10.3389/fmicb.2020.02028

**Published:** 2020-08-21

**Authors:** Jake C. Szamosi, Jessica D. Forbes, Julia K. Copeland, Natalie C. Knox, Shahrokh Shekarriz, Laura Rossi, Morag Graham, Christine Bonner, David S. Guttman, Gary Van Domselaar, Michael G. Surette, Charles N. Bernstein

**Affiliations:** ^1^Department of Medicine, McMaster University, Hamilton, ON, Canada; ^2^Department of Internal Medicine, University of Manitoba, Winnipeg, MB, Canada; ^3^IBD Clinical and Research Centre, University of Manitoba, Winnipeg, MB, Canada; ^4^National Microbiology Laboratory, Public Health Agency of Canada, Winnipeg, MB, Canada; ^5^Centre for the Analysis of Genome Evolution and Function, University of Toronto, Toronto, ON, Canada; ^6^Department of Medical Microbiology and Infectious Diseases, University of Manitoba, Winnipeg, MB, Canada; ^7^Department of Biochemistry and Biomedical Sciences, McMaster University, Hamilton, ON, Canada

**Keywords:** microbiome, standards, technical variability, 16S rRNA, intestinal biopsies, inflammatory bowel diseases, replicability

## Abstract

**Background:**

In studies evaluating the microbiome, numerous factors can contribute to technical variability. These factors include DNA extraction methodology, sequencing protocols, and data analysis strategies. We sought to evaluate the impact these factors have on the results obtained when the sequence data are independently generated and analyzed by different laboratories.

**Methods:**

To evaluate the effect of technical variability, we used human intestinal biopsy samples resected from individuals diagnosed with an inflammatory bowel disease (IBD), including Crohn’s disease (*n* = 12) and ulcerative colitis (*n* = 10), and those without IBD (*n* = 10). Matched samples from each participant were sent to three laboratories and studied using independent protocols for DNA extraction, library preparation, targeted-amplicon sequencing of a 16S rRNA gene hypervariable region, and processing of sequence data. We looked at two measures of interest – Bray–Curtis PERMANOVA *R*^2^ values and log2 fold-change estimates of the 25 most-abundant taxa – to assess variation in the results produced by each laboratory, as well the relative contribution to variation from the different extraction, sequencing, and analysis steps used to generate these measures.

**Results:**

The *R*^2^ values and estimated differential abundance associated with diagnosis were consistent across datasets that used different DNA extraction and sequencing protocols, and within datasets that pooled samples from multiple protocols; however, variability in bioinformatic processing of sequence data led to changes in *R*^2^ values and inconsistencies in taxonomic assignment and abundance estimates.

**Conclusion:**

Although the contribution of DNA extraction and sequencing methods to variability were observable, we find that results can be robust to the various extraction and sequencing approaches used in our study. Differences in data processing methods have a larger impact on results, making comparison among studies less reliable and the combined analysis of bioinformatically processed samples nearly impossible. Our results highlight the importance of making raw sequence data available to facilitate combined and comparative analyses of published studies using common data processing protocols. Study methodologies should provide detailed data processing methods for validation, interpretability, reproducibility, and comparability.

## Introduction

The human microbiome – the community of microorganisms that live on and within the human body – is increasingly recognized as playing a pivotal role in health and disease. A substantial body of evidence indicates that the composition of the microbiome can be both a driver and a marker of health status (Hollister et al., 2014). The link between the gut microbiome and diseases of the gastrointestinal tract such as inflammatory bowel disease (IBD) is especially well established (Matsuoka and Kanai, 2015). Treatment with antibiotics is known to improve IBD symptoms in some patients with specific types of IBD (Khan et al., 2011), and previous research has shown that IBD is associated with a decrease in bacterial diversity, a reduction in the phylum Firmicutes, and an increase in the phylum Proteobacteria (Matsuoka and Kanai, 2015). However, associations reported for IBD and bacterial taxa at the genus and species level have been inconsistent (Matsuoka and Kanai, 2015). The dynamic nature of the gut microbiome and the panoply of confounding factors that can influence its structure have frustrated our ability to resolve these inconsistencies (Lozupone et al., 2012). Of these, the technical variability arising from the lack of adherence to any standard approach for studying the microbiome is strongly implicated in the inconsistency of the results reported by studies investigating the association between gut microbiome composition and disease status (Wesolowska-Andersen et al., 2014; Choo et al., 2015; Thomas et al., 2015; Gerasimidis et al., 2016; Gohl et al., 2016; Costea et al., 2017).

In order to corroborate and validate which aspects of the microbiome associate with health and disease, results and conclusions need to be comparable across studies. Variability in methodology, however, can potentially change the detected community structure and influence the interpretation of the results. Such variability constitutes a serious obstacle to replicating study findings or drawing conclusions beyond the scope of each individual study. Methodological variability can be introduced at all stages of a microbiome research project, including sample collection, transport, processing, and storage; DNA extraction; the region and primers used for amplification; data processing; analysis methods; and interpretation of results (Santiago et al., 2014; Wesolowska-Andersen et al., 2014; Choo et al., 2015; Thomas et al., 2015; Gerasimidis et al., 2016; Gohl et al., 2016; Shaw et al., 2016; Song et al., 2016; Costea et al., 2017).

Prior studies have evaluated how methodological differences influence the results of microbiome assays (Thomas et al., 2015). Several studies based on 16S amplicon sequencing of stool specimens have shown that community composition is typically robust to extraction method (Santiago et al., 2014; Gerasimidis et al., 2016; Gohl et al., 2016; Shaw et al., 2016; Costea et al., 2017). Conflictingly, a number of studies focusing on shotgun metagenomics assays report that apparent taxon relative abundances were sensitive to the method used for DNA extraction (Wesolowska-Andersen et al., 2014; Jones et al., 2015; Wu et al., 2019). Multicentre studies assessing technical variability of 16S rRNA sequencing for microbiome analysis have reported its contribution to be significant, despite high intra-center reproducibility (Hiergeist et al., 2016). To address a lack of standard protocols, initiatives including International Human Microbiome Standards (IHMS) and the Microbiome Quality Control (MBQC) project have been developed to promote reproducibility in microbiome research and help inform study design to reduce inter-study technical variability (Santiago et al., 2014; Sinha et al., 2015; Hayakawa et al., 2018). Their findings indicate that the choice of DNA extraction protocol and amplicon primer sets substantially influence inter-laboratory variability (Jones et al., 2015; Sinha et al., 2015; Hayakawa et al., 2018). Other studies have also shown that both amplicon-based microbiome surveys and shotgun metagenomics methodologies suffer from biases due to a wide range of factors, including primer bias and library preparation (Jones et al., 2015; Sinha et al., 2015; Gohl et al., 2016; Hayakawa et al., 2018). As well, the choice of bioinformatic processing pipeline used to convert DNA sequences into count tables can impact the characterization of the microbial community (Golob et al., 2017). Therefore, development of best practices for human microbiome research will allow for increased reproducibility and comparability among studies.

In the present study, three participating laboratories performed 16S rRNA gene amplicon sequencing of matched samples of intestinal mucosal microbiota from 32 individuals using the methods favored by each lab. We assessed the effects of technical variability at three key stages of data generation: (1) genomic DNA extraction, (2) amplification and sequencing of the 16S rRNA gene hypervariable region, and (3) bioinformatic processing of reads into count tables. We report our findings here along with a discussion of the implications our results have on conducting multicentre studies of the gut microbiome in gastrointestinal health and disease.

## Materials and Methods

### Study Design

The Research Ethics Board at the University of Manitoba approved this study.

**FIGURE 1:**
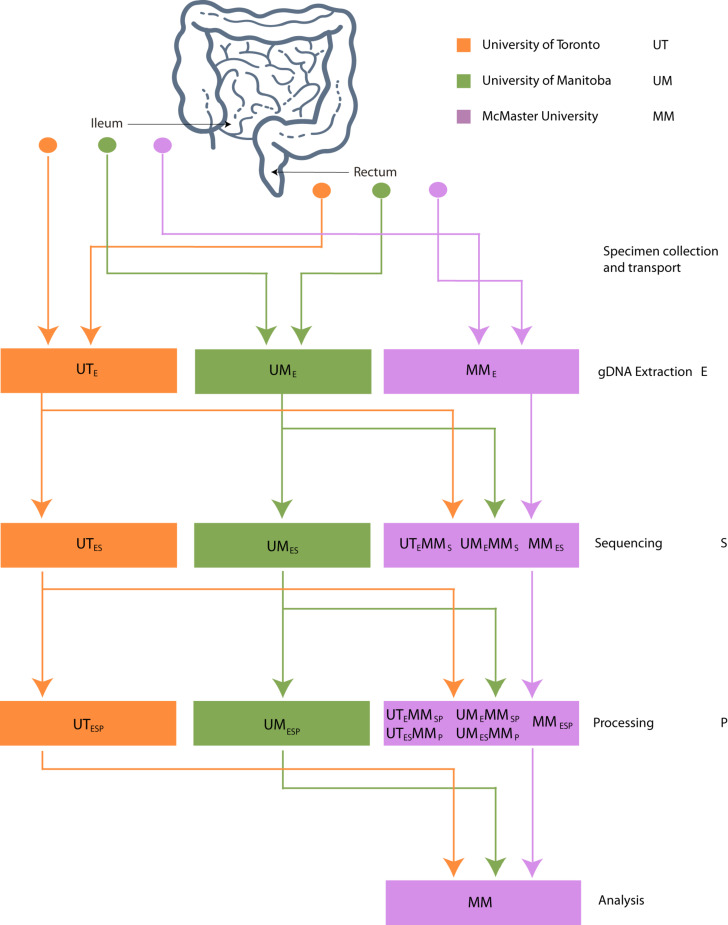
Schematic of experimental design. Biopsies taken from 32 patients were sent to 3 centers for DNA extraction. DNA was independently sequenced, extracted, and processed at each center and sent to McMaster (MM) for sequencing. FASTQ files were processed into OTU or ASV tables on site and sent to MM for processing.

The datasets generated from 16S rRNA gene amplicon sequencing experiments must, by necessity, proceed though stages of DNA extraction (E), sequencing (S), and bioinformatic processing (P). To investigate the effect of intercentre technical variability introduced at each of these stages, the extracted DNA, sequence data, and bioinformatically processed data independently generated at each study center must at each stage be redirected to a single, designated lab and then proceed through the remaining stages. This is reflected in our methodological study design, which we describe here and represent schematically in Figure 1.

Three Canadian research laboratories participated in this assessment: Center for The Analysis of Genome Evolution and Adaptation at the University of Toronto in Toronto, ON (UT); Public Health Agency of Canada’s National Microbiology Laboratory in collaboration with the University of Manitoba in Winnipeg, MB (UM); and the Farncombe Metagenomics Facility and Surette laboratory at McMaster University in Hamilton, ON (MM). Thirty-two participants were recruited for this study. Twenty-two participants had inflammatory bowel disease (IBD) – Crohn’s disease (CD; *n* = 12) or ulcerative colitis (UC; *n* = 10). Ten were non-IBD controls – irritable bowel syndrome (*n* = 2), collagenous colitis (*n* = 1), or healthy (*n* = 7). The ten non-IBD controls were grouped together for the purpose of this analysis. Tissue from six adjacent biopsy specimens were collected from the rectum and the ileum (or cecum) of each subject (*N* = 384). All biopsies were placed in a cryovial and immediately stored in liquid nitrogen until same-day transfer to −80°C. Two replicates from each biopsy site were pooled and shipped on dry ice to each of the three participating labs for DNA extraction (UM_E_, UT_E_, MM_E_), sequencing (UM_ES_, UT_ES_, MM_ES_), and bioinformatic processing (UM_ESP_, UT_ESP_, MM_ESP_) to produce amplicon sequence variant (ASV) or operational taxonomic unit (OTU) count tables using each lab’s independent protocols without regard for the methods used by the other participating labs. To evaluate variability introduced at the DNA extraction step, aliquots of extracted DNA from UM and UT were shipped to MM, where they were amplified, sequenced, and data analyzed by the MM laboratory (UM_E_MM_SP_, UT_E_MM_SP_). To assess the variability introduced at the data processing step, sequence data from all centers were also processed together into a single taxon count table by MM (UM_ES_MM_P_, UT_ES_MM_P_). Samples that stayed at a single institution from one phase of processing to the next were not subjected to “mock shipping” of any kind. Finally, all taxon count tables were analyzed separately and together to evaluate the degree of variability introduced in each of the extraction, sequencing, and bioinformatic processing steps. An overview of the data sets can be found in Table 1.

**TABLE 1:** Dataset description.

	Data Set^a,b^	DNA Extraction	Sequencing	Bioinformatic Processing	Sample Count	Min Depth	Max Depth	ASV/OTU^*c*^ Count
**Unpooled**	MM_ESP_	MM	MM	MM	59	1,029	32,305	400
	UT_ESP_	UT	UT	UT	64	1,022	167,922	757
	UT_ES_MM_P_	UT	UT	MM	58	1,125	114,877	396
	UT_E_MM_SP_	UT	MM	MM	61	2,745	33,415	403
	UM_ESP_	UM	UM	UM	64	5,480	273,886	289
	UM_ES_MM_P_	UM	UM	MM	62	1,963	103,781	418
	UM_E_MM_SP_	UM	MM	MM	60	1,445	24,413	411
**Pooled**	PE	All	MM	MM				
	PS	All	All	MM				
	PP	All	All	All				

*^*a*^E, Extraction; S, Sequencing; P, Bioinformatic Processing. ^*b*^PE, pooled post-extraction; PS, pooled post-**s**equencing; PP, pooled post-bioinformatic processing. All pooled data sets consist of samples randomly drawn from unpooled data sets. ^*c*^ASV, Amplicon Sequence Variant; OTU, Operational Taxonomic Unit.*

### Genomic DNA Extraction

#### University of Toronto

Biopsy specimens were thawed on ice and genomic DNA was extracted using the MO BIO PowerSoil DNA Isolation Kit (Qiagen) according to manufacturer’s instructions. The samples were homogenized in Power Bead tubes for 3 min in a bead beater and DNA was eluted in 100 μL of elution buffer.

#### University of Manitoba

Biopsy specimens were thawed at room temperature. Genomic DNA was isolated using the MO BIO PowerSoil DNA Isolation Kit with Sunagawa modifications (Baker and Kellogg, 2014), as follows: To increase the yield and quality of DNA preparations, we modified the manufacturer’s instructions by: (1) adding 0.19 μL lysozyme (Epicenter; final: 10 U μL-1) to the Bead Solution/sample mixture, followed by an incubation of 10 min at room temperature; (2) adding 25 μL proteinase K (Invitrogen; final: ∼0.8 mg mL-1) to the lysozyme-treated mixture, followed by an incubation for 60 min at 65°C; and (3) adding 400 mg of each 0.1 and 0.5 mm zirconia/silica beads before samples were homogenized by bead beating.

#### McMaster

Genomic DNA was isolated according to the method described in Stearns et al. (2015), with modifications. Briefly, each biopsy was transferred to a screw cap tube containing 2.8 mm ceramic beads, 0.1 mm glass beads, GES solution, and sodium phosphate buffer. Samples were mechanically lysed in a homogenizer for 3 min at 3000 rpm for 2 cycles. Samples were then incubated at 37°C for 1 h after the addition of lysozyme and RNaseA (mutanolysin was excluded), followed by a second incubation at 65°C for 1 hr after the addition of SDS, NaCl and Proteinase K. Samples then underwent centrifugation at 13000 rpm for 5 min. The supernatant was then added to equal volume of phenol:chloroform:isoamyl for further extraction and purification. The samples were vortexed and again centrifuged at 13000 rpm for 10 min. The top aqueous layer was then mixed with 200 μL of DNA binding buffer as part of the Zymo DNA Clean and Concentrator-25 kit. The DNA was purified as per the kit instructions and finally eluted with 50 μL of ultrapure water.

### Library Preparation and Sequencing

#### University of Toronto

The V4 hypervariable region of the 16S rRNA gene was amplified using a universal 515F (GTGCCAGCMGCCGCGGTAA) forward sequencing primer and a uniquely barcoded 806R (GGACTACHVGGGTWTCTAAT) reverse sequencing primer to allow for multiplexing (Caporaso et al., 2012). Amplification reactions were performed using 12.5 μL of KAPA2G Robust HotStart ReadyMix (KAPA Biosystems), 1.5 μL of 10 μM forward and reverse primers, 9.5 μL of sterile water, and 1 μL of DNA. The V4 region was amplified by cycling the reaction at 95°C for 3 min, 27 cycles of 95°C for 15 s, 50°C for 15 s, and 72°C for 15 s, followed by a 5-min extension at 72°C. All amplification reactions were done in triplicate, visualized on a 1% agarose Tris/Borate/EDTA (TBE) gel, and then pooled to reduce amplification bias. Pooled triplicates were quantified using the Qubit HS DNA kit and combined in equal concentrations. The final library was gel purified on a 2% agarose tris-acetate-EDTA (TAE) gel due to the presence of dual bands after amplification. The gel-extracted library was then purified using 1.8× magnetic Ampure XP beads and quantified. Approximately 7 pM of the purified library pools and PhiX spike-in control DNA (at 5%) were loaded on to the Illumina MiSeq for sequencing, according to manufacturer instructions (Illumina, San Diego, CA, United States). Sequencing was performed on 64 samples in a single run using the V2 chemistry (300 cycles; 2 × 150 bp).

#### University of Manitoba

Templates were prepared following the Illumina protocol for 16S rRNA gene amplicons for the MiSeq platform with modifications described elsewhere (Alfa et al., 2018). Briefly, the 16S rRNA gene V4 region was amplified using primers 515fXT (GTGBCAGCMGCCGCGGTAA) and 806rXT (GGACTACHVGGGTWTCTAAT). Amplification was performed in triplicate using 12.5 μL of KAPA2G Robust HotStart ReadyMix (KAPA Biosystems), 5 μL each of 1.0 μM forward and reverse primers, 9.5 μL of sterile water, and 2.5 μL of DNA. The V4 region was amplified by cycling at 95°C for 3 min, 25 cycles of 95°C for 30 s, 55°C for 30 s, and 72°C for 30 s, followed by a 5-min extension at 72°C. Triplicate reactions were pooled and purified by AMPureXP. Quality control, quantification, normalization, pooling, and sequencing of the library was performed according to Illumina instructions, and approximately 11 pM of the pools and 37.5% PhiX spike-in control DNA were used for sequencing using the V3 chemistry (600 cycles; 2 × 300 bp). A single Illumina MiSeq run was performed on 65 multiplexed samples including a no-template control (NTC) and mock community of known composition (HM-782D; BEI Resources, Manassas, VA, United States).

#### McMaster University

Purified DNA was used to amplify the hypervariable regions V3 and V4 of the 16S rRNA gene using a two-stage nested PCR approach. Initially the 8f (AGAGTTTGATCCTGGCTCAG) to 926r (CCGTCAATTCCTTTRAGTTT) region of the 16S rRNA gene was amplified using 100 ng of template with 1 unit of Taq, 1× buffer, 1.5 mM MgCl_2_ (ThermoFisher *Taq* DNA recombinant kit), 0.4 mg/mL bovine serum albumin (BSA; Sigma), 0.2 mM dNTPs (New England BioLabs), and 10 pM of each primer. The reaction was carried out at 98°C for 5 min followed by 15 cycles of 98°C for 30 s, 56°C for 30 s and 72°C for 60 s, with a final extension of 72°C for 10 min. The triplicate reaction was then recombined and used as template in the second stage of PCR. A total of 3 μL of the first pooled reaction was used as template for PCR using 1 unit of Taq, 1× buffer, 1.5 mM MgCl_2_, 0.4 mg/mL BSA, 0.2 mM dNTPs, and 5 pM of 341F (CCTACGGGAGGCAGCAG) and 806R (GGACTACHVGGGTWTCTAAT) Illumina adapted primers, as described elsewhere (Bartram et al., 2011). The reaction was carried out at 98°C for 5 min followed by 25 cycles of 98°C for 30 s, 50°C for 30 s and 72°C for 30 s, with a final extension of 72°C for 10 min. The resulting PCR products were visualized on a 1.5% agarose gel. Positive amplicons were normalized using the SequalPrep normalization kit (ThermoFisher) and sequenced with the Illumina MiSeq platform across seven runs. Spike-in was either 5% PhiX or 1% PhiX plus 2.5–19% bacterial genomic DNA from other experiments.

A comparison of the extraction, library preparation, and sequencing methods among the three centers can be found in Table 2.

**TABLE 2:** Extraction and sequencing methods.

Step		UT	UM	MM
Extraction	Mechanical lysis	MO BIO PowerSoil DNA Isolation kit standard protocol	MO BIO PowerSoil DNA Isolation kit + zirconia/silica beads during homogenization	Bead-beating with ceramic and glass beads in GES + sodium phosphate
	Enzymatic lysis		MO BIO PowerSoil DNA isolation kit + lysozyme incubation + proteinase K	Lysozyme and RNaseA
	Incubation			SDS, NaCl, Proteinase K
	Extraction		MO BIO PowerSoil DNA isolation kit	-centrifugation -phenol, chloroform, isoamyl
	Purification			Zymo DNA Clean and Concentrator-25 kit
Amplification	Primers	515F and 806R primers; V4 region	515fXT and 806rXT primers; V4 region	Nested PCR: 8f and 926r primers [first reaction (1)] and 341f and 806r primers; V3-V4 region [second reaction (2)]
	PCR Mix	−12.5 μL KAPA2G Robust HotStart ReadyMix (KAPA Biosystems) −1.5 μL of 10 μM each primer −9.5 μL of sterile water −1 μL of template DNA	−12.5 μL of KAPA2G Robust HotStart ReadyMix (KAPA Biosystems) −5 μL of 1.0 μM each primer −9.5 μL of sterile water −2.5 μL of template DNA. The V4 region was amplified by cycling at	−1 unit of Taq, 1× buffer, 1.5 mM MgCl_2_ (ThermoFisher) −0.4 mg/mL bovine serum albumin −0.2 mM dNTPs −10 pM of each primer (1) **or** 5pM of each primer (2) −100 ng of template DNA (1) **or** 3 μL of first reaction product (2)
	PCR Cycles	−3 min @ 95°C −27× −15 s @ 95°C −15 s @ 50°C −15 s @ 72°C −5 min @ 72°C.	−3 min @ 95°C −25× −30 s @ 95°C −30 s @ 55°C −30 s @ 72°C −5 min @ 72°C	−5 min @ 98°C −15 × (1) **or** 25 × (2) −30 s @ 98°C −30 s @ 56°C −60 s (1) **or** 30 s (2) @ 72°C −10 min @ 72°C
Library Prep and Sequencing	Purification and Normalization	-gel purification on 2% TAE −1.5× Ampure XP magnetic beads	-purified by AMPure XP -quantitated with PicoGreen and pooled in equimolar amounts	SequalPrep normalization kit (ThermoFisher)
	Sequencing	-Illumina MiSeq; V2 chemistry −5% PhiX spike-in −2 × 150bp	-Illumina MiSeq; V3 chemistry −37.5% PhiX spike-in −2 × 300bp	-Illumina MiSeq; V2 chemistry -spike-in either 5% PhiX alone or 1% PhiX and 2.5% – 19% bacterial genomic DNA −2 × 250bp

### Bioinformatic Processing

#### University of Toronto

The UNOISE pipeline, available through USEARCH v.10.0.240 (Edgar, 2010, 2013; Bartram et al., 2011), was used to generate an OTU count table. The last base was removed from all sequences. Paired-end sequences were merged using *– fastq_mergepairs* with a *– fastq_maxee* of 1.0 to remove poor quality reads (Edgar, 2016). Reads were quality trimmed using *– fastq_filter* with a *– fastq_maxee* of 0.5. Merged sequence pairs shorter than 233 base pairs were discarded. The remaining merged pairs were de-replicated. Sequences were denoised and chimeras were removed using the *unoise3* command. Assembled sequences were then mapped back to the chimera-free, denoised sequences at 97% identity to generate OTUs. Taxonomy assignment was performed using the SINTAX algorithm implemented in USEARCH with the UNOISE compatible Ribosomal Database Project v.16 database (RDP) (Wang et al., 2007) at minimum 80% bootstrap confidence threshold. OTU sequences were aligned using the PyNast aligner in QIIME (Caporaso et al., 2010). Sequences that did not align were discarded.

#### University of Manitoba

Amplicon data was processed using the mothur software suite v.1.38.0 (Schloss et al., 2009). Paired-end reads (2 × 300 bp) were assembled into contigs using Needleman–Wunsch pairwise alignments and V4 amplicon primers were removed. Contigs were removed if they were >275 bp in length, contained homopolymers longer than 8 bp, or contained any ambiguous base calls or chimeric artifacts (via UCHIME) (Edgar et al., 2011). A custom reference alignment specific to the sequenced 16S rRNA gene V4 region was created by trimming the full length 16S rRNA gene SILVA v.128 reference alignment to the region of interest and aligning contigs to the trimmed reference database (Pruesse et al., 2007); contigs aligning outside of the 16S rRNA gene V4 region were removed. To account for sequencing error, contigs that differed by a maximum of 2 bp were clustered together and considered the same sequence. The co-sequenced mock community was selected from the data to assess sequence error rates from the Illumina MiSeq run. The mock community was excluded from downstream analyses. Taxonomic classification was performed with the mothur software suite (Cole et al., 2009) using a naive Bayesian classifier implementing the *k*-nearest neighbor algorithm adapted from Wang et al. (2007) with the RDP v.16 training set (Cole et al., 2009) at 80% bootstrap confidence. Sequences classified as unwanted lineages, such as chloroplast, mitochondria, archaea, eukaryota, or unknown, were subsequently removed. Sequences were binned into species-level OTUs using the average neighbor algorithm at a ≥97% sequence similarity. The OTUs were taxonomically classified using the RDP reference database with an 80% minimum bootstrap confidence threshold.

#### McMaster University

Cutadapt v.1.14 (Martin, 2011) was used to filter and trim adapter sequences and PCR primers from the raw reads using a quality score cut off of 30 and a minimum read length of 100 bp. After trimming, the reverse Illumina reads were too short to merge with the forward reads, so only the forward reads, corresponding to the V4 region, were used. Sequence variants were then resolved from the trimmed raw reads using DADA2 (Callahan et al., 2016) as follows: DNA sequences were filtered and trimmed based on the quality of the reads for each Illumina run separately, error rates were learned separately for each run, and sequences were denoised to produce ASV count tables.

The sequence variant tables from the different Illumina runs were merged to produce a single ASV table. Bimeras were removed and taxonomy was assigned using the DADA2 implementation of the RDP classifier against the SILVA v.1.2.8 database (Pruesse et al., 2007), at 50% bootstrap confidence.

DADA2 was also used to bioinformatically process the sequence data generated from the UM and UT study centers. The FASTQ files generated at each study center were merged together to create three combined datasets (UM_S_, UT_S_, and MM_S_). Primer sequences were removed as necessary using Cutadapt. Merged and trimmed reads were then used as input into DADA2. The MM_S_ sequences were trimmed to the same length and region as the UT_S_ and UM_S_ sequences using DADA2 after denoising so the ASV tables could be merged.

Samples sequenced at UT were processed as paired-end sequences with *filterandTrim()* cutoffs of length = (150,150), maxEE = (2,5), and *q* = 11. Samples sequenced at UM were processed as paired-end sequences with *filterandTrim()* cutoffs of length = (150,150), maxEE = (2,5), and *q* = 11. Samples sequenced at MM were processed as single-end sequences using only R1, since the reverse read was of too poor quality to use. The *filterandTrim()* cutoffs were length = 220, maxEE = 2, and *q* = 25. After running DADA2 separately for each Illumina run, the reverse complement was taken of sequences from UT and UM to match sequences from MM, and all sequences were trimmed to a length of 220 bp. ASVs were estimated per-sample and merged.

Once ASV tables were generated for each study center, the tables were merged, filtered for chimeras, and taxonomies assigned as described above. Sequence variants were filtered to remove all non-bacterial reads (this includes any reads assigned to kingdom *Eukaryota*, those lacking a phylum assignment, and those assigned to family *Mitochondria*). We also removed any sequence variants present only once in the dataset.

After this filtering, samples with fewer than 1,000 reads were eliminated and sequence variants were re-filtered to remove any variants whose mean abundance across the whole dataset was less than 0.01%.

### Final Datasets

Our experimental design generated seven unpooled datasets (Table 2 and Figure 1). Of these seven, three were independently extracted, sequenced, and bioinformatically processed at each study center (UM_ESP_, UT_ESP_, MM_ESP_), so that they differed at all three levels of variability. Two were generated from DNA independently extracted and sequenced from UM and UT, respectively, and then processed at MM (UM_ES_MM_P_, UT_ES_MM_P_), so that they, together with MM_ESP_, differed at the levels of extraction and sequencing, but not bioinformatic processing. Finally, two were generated from DNA independently extracted at UM and UT, respectively, and then sequenced and processed at MM (UM_E_MM_SP_, UT_E_MM_SP_), so that they, together with MM_ESP_, differed at the level of extraction only. Each of these seven datasets was analyzed separately, and the comparison of these analyses is intended to simulate comparison of the results of different studies, or of data produced by different studies.

We additionally constructed three sets of pooled datasets. Samples were randomly drawn from the seven unpooled datasets such that each sample was represented once in each pooled dataset, and the two samples from a given patient both originated from the same unpooled dataset. The pooled-extraction (P_E_) dataset included samples drawn from the UM_E_MM_SP_, UT_E_MM_SP_, and MM_ESP_ datasets. The pooled-sequencing (P_S_) dataset included samples drawn from the UM_ES_MM_P_, UT_ES_MM_P_, and MM_ESP_ datasets. Finally, the pooled-processing (P_P_) dataset included samples drawn from the UM_ESP_, UT_ESP_, and MM_ESP_ datasets. We randomly generated each type of pooled dataset 1,000 times in order to get a range of estimates for our parameters of interest. These pooled datasets are intended to assess the validity of co-analysis of samples originating from different studies. The P_E_ and P_S_ datasets were combined at the level of ASVs while the P_P_ dataset was combined at the level of genus.

**FIGURE 2:**
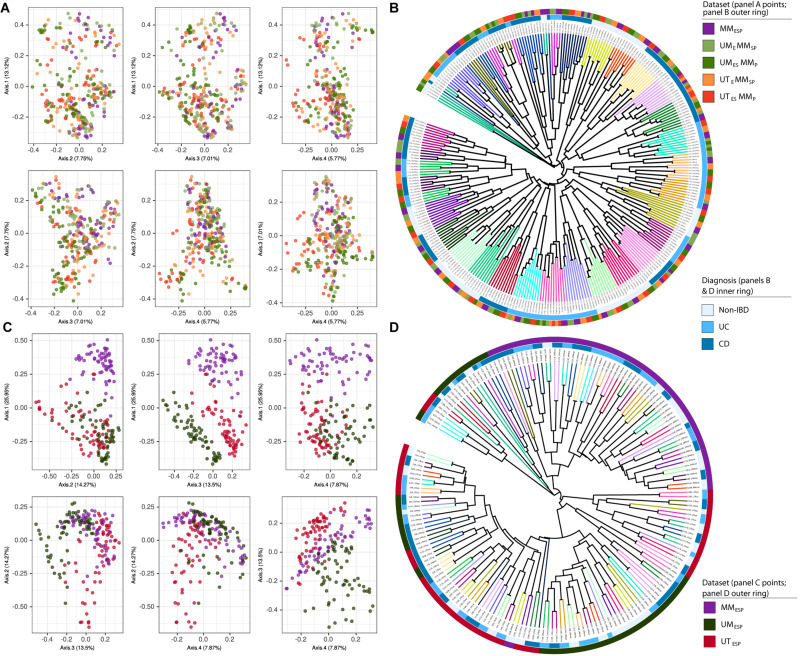
Samples independently extracted and sequenced at each study center, bioinformatically processed at McMaster, and visualized together as **(A)** PCoA plots and **(B)** UPGMA trees. Samples independently extracted, sequenced, bioinformatically processed at each study center, and visualized as **(C)** PCoA plots and **(D)** UPGMA trees based on Bray-Curtis dissimilarity. Tree branches are colored according to patient. Diagnosis is indicated by the inner colored ring. Dataset is indicated by the outer colored ring.

Finally, we constructed two supersets of all the samples from all seven unpooled datasets. These were used only to generate the ordinations and UPGMA trees shown in Figure 2. The first superset contained all samples bioinformatically processed at McMaster (all datasets except UM_ESP_ and UT_ESP_) and was combined at the ASV level (Figures 2A,B), and the second superset contained the samples that differed at all three levels of variation (UM_ESP_, UT_ESP_, and MM_ESP_) and was combined at the genus level (Figures 2C,D).

### Statistical Analysis

All downstream analysis was conducted in the R v.3.4.4 statistical programming language (R Core Team, 2018). We curated the data and generated plots using *phyloseq v.1.22.3* (McMurdie and Holmes, 2013) and the following *tidyverse* (Wickham, 2017) packages: *dplyr v.0.7.6*, *tidyr v.0.8.1*, *rlang v.0.2.1*, and *ggplot2 v.3.0.0* (Wickham, 2009). Color palettes used in the figures were adapted from *RColorBrewer v.1.1.2* (Neuwirth, 2014) with additional colors generated using the I Want Hue color tool (Médialab).

To visualize sample distances and identify sample clustering, we calculated and ordinated Bray–Curtis dissimilarities using *phyloseq*. Principal coordinate analysis (PCoA) plots were generated using *phyloseq* and *ggplot2*. Unweighted pair group method with arithmetic mean (UPGMA) trees based on Bray–Curtis dissimilarities were generated using the *ape v.5.2* (Paradis and Schliep, 2018) package and the *hclust()* function in R. Trees were visualized using the *stringi v.1.2.3* (Gagolewski, 2018) package in R and the Interactive Tree of Life (iTOL) v.3 (Letunic and Bork, 2016).

**FIGURE 3:**
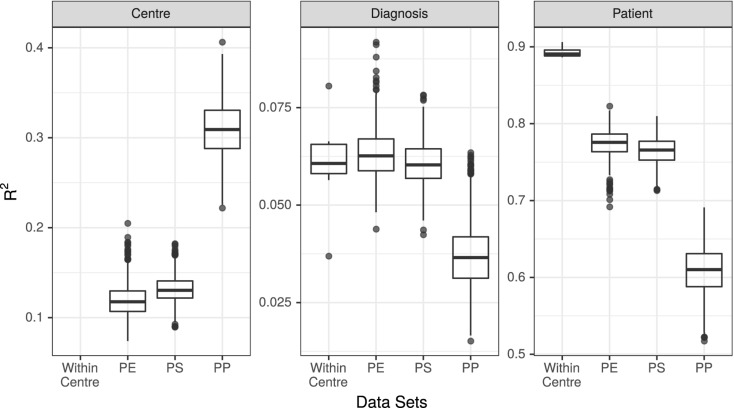
*R*^2^ values associated with center, diagnosis, and patient in all 7 unpooled datasets and 3 pooled datasets. PE, Post-Extraction; PS, Post-Sequencing; PP, Post-Processing.

The variability in the microbiota attributable to *study center* (at the stages of extraction, sequencing, and bioinformatic processing), *diagnosis*, and *patient* was evaluated by PERMANOVA using Bray–Curtis dissimilarities implemented in the *adonis()* function in *vegan v.2.5.2* (Dixon, 2003; Oksanen et al., 2018; Figure 3) as follows: *adonis(bray_dist* ∼ *Centre*^1^ + *Diagnosis* + *Patient, by* = *“margin”)*. Bray–Curtis dissimilarities were calculated based on ASV or OTU relative abundance (McMurdie and Holmes, 2014) for all datasets except the pooled PP datasets. Because independently generated OTUs cannot be compared, Bray–Curtis dissimilarities in the pooled PP datasets were calculated based on genus-level relative abundances instead.

Differential abundances among the three diagnosis groups (UC, CD, and non-IBD) of the 25 genera with the highest mean abundance were assessed on raw counts using a negative binomial linear mixed model implemented in *glmmTMB v.0.2.2* (Brooks et al., 2017) with a random intercept for *patient* as follows: *glmmTMB(taxon_abundance* ∼ *Diagnosis* + *offset(log(libsize))* + *(1| Patient), family* = *“nbinom1”)*. We corrected for multiple tests using a false discovery rate (FDR) of 5% and *n* = 25 (for the 25 genera).

## Results

### Ordination of All Samples

In the ordination of the superset of all five unpooled datasets bioinformatically processed at McMaster, there was no apparent clustering by extraction or sequencing method in the PCoA plot (Figure 2A), and the UPGMA tree showed strong clustering by patient only (Figure 2B).

The ordination of the superset of the three datasets bioinformatically processed at different study centers showed strong clustering by study center (Figure 2C). The MM_ESP_ samples separated from the UT_ESP_ and UM_ESP_ samples on the first axis (27%) and the UT_ESP_ and UM_ESP_ samples separated from each other on the fourth axis (8%). This clustering is also visible in the UPGMA tree constructed for these samples (Figure 2D), where patient clusters are stratified across study centers. The separation by center appears to arise from multiple steps in the bioinformatic pipeline, and not simply from the fact that the different centers used different databases to assign taxonomy. Figure S2 shows the ordination and clustering of this same superset after re-assigning taxonomy to the UT_ESP_ data using the same database as MM_ESP_ (SILVA). The separation between UT_ESP_ and MM_ESP_ samples is weaker than in Figures 2C,D, but still largely maintained.

### Comparison of Within-Center Analyses

In order to explore the validity of comparing results among studies, we analyzed each unpooled dataset individually and compared the results. Specifically, we conducted PERMANOVA tests and tests of differential abundance on the seven unpooled datasets.

The results of the PERMANOVA test were broadly consistent across all unpooled datasets, with *R*^2^ values for *diagnosis* ranging from 3.7% (UM_ESP_) to 8.1% (UT_ES_MM_P_), with a mean of 6.1%. The *R*^2^ values for *patient* ranged from 89% (MM_ESP_) to 91% (UT_ESP_), with a mean of 89%. All *p*-values associated with diagnosis and patient were <0.001 (Figure 3 “Within Center”).

**FIGURE 4:**
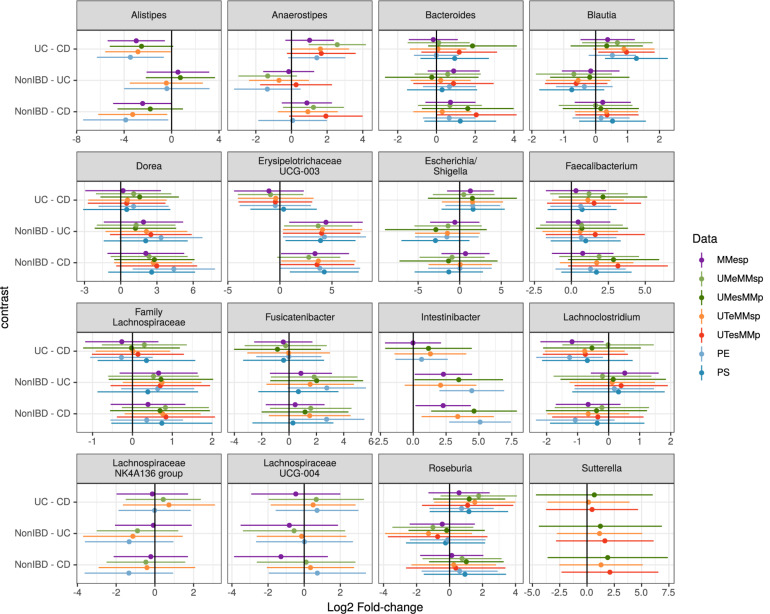
Estimated log2 fold-changes (with 95% confidence intervals) of the 16 most-common genera for all three diagnosis pairs in all 5 MMp datasets and the pooled PE and PS datasets.

Choosing the 25 genera with the highest mean abundance in each unpooled dataset yielded 47 total genera. The five datasets that were bioinformatically processed at MM yielded 27 distinct genera, of which 16 were present in at least three of the five datasets. Although the differential abundance among diagnoses was not statistically significant in any of the top 25 taxa in any of the datasets when assessed by generalized linear mixed model, the estimated fold changes and confidence intervals were similar among datasets (Figure 4). The 25 most-abundant genera from three datasets generated at the different study centers (MMESP, UT_ESP_, and UM_ESP_) yielded 44 distinct genera, of which 16 were present in at least two of the three datasets. These 16 genera showed similar estimated fold changes (Supplementary Figure S1).

### Evaluation of Intracentre Variability

We conducted PERMANOVA tests on all 3,000 pooled datasets (1,000 each of P_E_, P_S_, and P_P_). The mean *R*^2^ value associated with *diagnosis* in the P_E_ datasets was 6.3%, with 95% of replicates yielding *R*^2^ values between 5.3 and 7.7%. The mean R^2^ of *diagnosis* was 6.1% (5.0–7.3%) in the P_S_ dataset, and 3.7% (2.3–5.6%) in the P_P_ dataset. The *R*^2^ values associated with *patient* were 77% (74–81%) in the P_E_ dataset, 76% (73–80%) in the P_S_ dataset, and 61% (55–67%) in the P_P_ dataset. Finally, the *R*^2^ value associated with *study center* was 12% (9–15%) in the P_E_ datasets, 13% (10–16%) in the P_S_ datasets, and 31% (25–37%) in the P_P_ datasets (Figure 3).

To assess the robustness of our taxon model estimates to this site variability, we used a single, randomly chosen replicate of each of the P_E_ and P_S_ datasets. We did not attempt this with a P_P_ dataset because it did not seem plausible that any study might attempt to test taxon differential abundance on a dataset that had not been bioinformatically processed together. As shown in Figure 4, the fold-change estimates in the pooled datasets can show higher variability than those from the original datasets but still demonstrate good agreement with those results.

## Discussion

In this study, we aimed to answer two distinct yet related questions: First, can different microbiome studies be meaningfully compared when they used different DNA extraction, sequencing, and bioinformatic processing methods? Second, can a single multicentre study be valid if the different centers use different methods at each step? We kept the extracted biopsies as similar as possible, in order to focus narrowly on the variation introduced by laboratory and bioinformatic methods.

We found that differences in our DNA extraction methods did not substantially affect the characterization of a microbial community (Figures 2A,B). Both our observed weak association with *diagnosis* and strong association with *patient* were robust to variability in extraction methods. Our findings are consistent with prior studies, although there is no clear consensus in the literature on the contribution of different extraction methods to technical variability. Extractions performed on fecal samples using the same methodology but on different dates or by different people have little apparent effect on the resultant 16S rRNA gene sequence data (Shaw et al., 2016). Comparisons of chaotropic, phenol/chloroform, and kit-based extraction methods of fecal samples similarly appear not to significantly contribute to observed technical variability in the resultant 16S rRNA gene sequence data (Gerasimidis et al., 2016). Extraction methods have, however, been reported to contribute significantly to variability in taxonomic classification from metagenomic data (Wesolowska-Andersen et al., 2014). Across all such studies, including our multicentre study, the contribution from extraction method to total variability was substantially outweighed by the biological variability targeted by the respective studies (Jones et al., 2015; Sinha et al., 2015; Gohl et al., 2016; Hayakawa et al., 2018).

Technical variability arising from variation in the methods used for 16S rRNA gene amplicon-based sequencing can be significant. Sources of this variability include amplicon primer bias (Sinha et al., 2015; Gohl et al., 2016; Teng et al., 2018), methods for library preparation (Jones et al., 2015; Gohl et al., 2016; Costea et al., 2017), and the 16S rRNA gene hypervariable region targeted for sequencing (Lozupone et al., 2012; Santiago et al., 2014; Hayakawa et al., 2018; Teng et al., 2018). In our study, two centers (UM and UT) independently chose to target the V4 hypervariable region, while the third (MM) targeted the longer V34 region. In either case, a diverse set of approaches were applied in the sequencing library preparation. The contribution of sequencing method to technical variability was detectable in our study, although, similar to our findings for the effect of extraction, its overall contribution to observed variability did not obscure the biological variable of interest.

We found that the biggest source of multicentre variation, and the one most likely to interfere with effective comparison of results or co-analysis of samples from different sources, was at the level of bioinformatic processing. The diversity of the various approaches chosen by each center was itself notable, with methods varying across all steps in the processing, including read trimming and cleaning, clustering or denoising methods, taxonomic assignment methods, and choice of taxonomic database. Although quality control and best practices were applied at each center for the bioinformatics processing of their datasets, there was little consistency across centers; this resulted in high internal reproducibility within each study center, but introduced significant variability that could have an appreciable impact on the interpretation of the resultant data, which we characterize in more detail below.

### Comparison of Individual Studies

We found that at the community level (Bray–Curtis PERMANOVA), unpooled results were comparable for both predictor variables. The percentage of variation attributed to *diagnosis* was small yet similar across study centers (3.7–8.1%), and the percent variation attributed to *patient* was large and similar across study centers (87–92%; Figure 3). This held true for all the examined stages of potential variability; however, the weaker variable (*diagnosis*) did show a larger change among single-method datasets when they varied in their bioinformatic processing (Figure 3, dark points).

When assessing taxon differential abundance, we found that estimated effect sizes (log2 fold-change) were comparable among unpooled datasets that varied in their extraction and sequencing methods (Figure 4); however, comparison among datasets that used different bioinformatic processing was less straightforward. No taxa present in the top 25 genera were common to all three datasets. Those that were present in the top 25 genera of at least two datasets did tend to have similar effect sizes (Supplementary Figure S1), but there appears to be more variation than in the datasets that used identical bioinformatic processing (Figure 4).

These results may help explain the many differences in scientific reports of microbiome results in health and disease from different laboratories. Although analyses are robust to variation in wet-laboratory methodology, differences in bioinformatic processing can generate different outcomes from duplicate specimens. This is particularly true of findings of taxon differential abundance, where even taxonomic classifications may not be consistent among studies. Our findings underscore the importance both of making raw sequence reads available and of providing detailed informatics methods, so that other researchers can more faithfully replicate them.

### Multicentre Studies

When samples that were extracted and sequenced at multiple centers were pooled and analyzed together (multi-method), the assessments of community-level differences were similar to those of the unpooled (single-method) datasets. The means of the percent of variation explained by *diagnosis* in the multi-method datasets were well within the range set by the single-method datasets. The percent of variation explained by *participant* was lower in the multi-method datasets than in the single-method datasets, but since it was near 90% in the single-center datasets, it is not surprising that it would drop when new potential sources of variability were introduced; it remained large and statistically significant in all P_E_ and P_S_ datasets. While the effect of *center* could be detected in these multi-center datasets, it did not detract substantially from our ability to detect either small or large community-level patterns. The estimated effect sizes of taxon differential abundances in the multi-center P_E_ and P_S_ datasets were also similar to those of the single-center datasets (Figure 4).

In datasets where samples were bioinformatically processed at multiple centers (P_P_), the effect on the output was more noticeable. The effect of *diagnosis* was statistically significant in all cases, but the mean diagnosis *R*^2^ value fell just at the bottom of the range set by our unpooled datasets, and 52% of the *R*^2^ values were lower than those estimated by any single-center analysis. The effect of *patient* dropped by a proportionally similar amount as in the P_E_ and P_S_ datasets, and the effect of center more than doubled compared to the P_E_ and P_S_ datasets (Figure 3). Taxon differential abundance could not be sensibly assessed in the P_P_ datasets since many high-abundance taxa were not consistent from sample to sample.

These results support the validity of multi-center studies. We find that researchers may be able to safely analyze data from samples that were extracted and sequenced in different locations using different protocols, as long as there is enough overlap in the 16S rRNA gene hypervariable region sequenced that the data can be informatically processed together. That said, pains should always be taken to identify any methodological biases, and it is ideal to use consistent protocols whenever possible. Additional variation could also arise from the inclusion of different patient populations at different centers, which was not assessed here.

## Conclusion

Although the contributions of DNA extraction and sequencing methods to variation were observable, we find that results can be robust to the various extraction and sequencing approaches used in our study. Differences in data processing methods have a larger impact on results, making comparison among studies less reliable and the combined analysis of bioinformatically processed samples nearly impossible. Our results highlight the importance of making raw sequence data available to facilitate combined and comparative analyses of published studies using common data processing protocols. Study methodologies should provide detailed data processing methods for validation, interpretability, comparability, and reproducibility.

## Data Availability Statement

The datasets presented in this study can be found in online repositories. The names of the repository/repositories and accession number(s) can be found below: https://www.ncbi.nlm.nih.gov/, PRJNA625750.

## Ethics Statement

The studies involving human participants were reviewed and approved by University of Manitoba Research Ethics Board. The patients/participants provided their written informed consent to participate in this study.

## Author Contributions

CNB, MS, DG, and GV developed the concept for the study. CNB collected the study samples. JC, LR, and CNB performed the wet-laboratory work. SS, JF, NK, JC, LR, and JS contributed to the bioinformatic processing. JS performed the statistical analysis. JS, JF, NK, JC, and GV wrote the manuscript. JS, JF, NK, JC, MS, CNB, DG, MG, and GV provided intellectual contribution. All authors read and improved the final manuscript.

## Conflict of Interest

The authors declare that the research was conducted in the absence of any commercial or financial relationships that could be construed as a potential conflict of interest.

## Footnotes

1 If samples were processed in different locations

## Supplementary Material

FIGURE S1 Estimated log2 fold-changes (with 95% confidence intervals) of the most common genera for all three diagnosis pairs in the UT_ESP_, UM_ESP_, and MM_ESP_ datasets.

FIGURE S2 Samples independently extracted, sequenced, and bioinformatically processed at each study center, except that the UT_ESP_ samples were assigned taxonomy using the same database as the MM_ESP_ samples (SILVA). Visualized as **(A)** PCoA plots and **(B)** a UPGMA tree based on Bray-Curtis dissimilarity. Tree branges are colored according to patient. Diagnosis is indicated by the inner colored ring. Dataset is indicated by the outer colored ring.
